# Stimuli-Responsive Protein Hydrogels: Their Design, Properties, and Biomedical Applications

**DOI:** 10.3390/polym15244652

**Published:** 2023-12-08

**Authors:** Yuxuan Lu, Yuhe Chen, Yuhan Zhu, Jingyi Zhao, Ketong Ren, Zhao Lu, Jun Li, Ziyang Hao

**Affiliations:** 1 School of Basic Medical Sciences, Capital Medical University, Beijing 100069, China; luyuxuan@mail.ccmu.edu.cn (Y.L.); chenyuhe2111057@mail.ccmu.edu.cn (Y.C.); 2 School of Pharmaceutical Sciences, Capital Medical University, Beijing 100069, China; zyh2124009@mail.ccmu.edu.cn (Y.Z.); zhaojingyi@mail.ccmu.edu.cn (J.Z.);

**Keywords:** protein-based hydrogels, stimuli-responsive, cross-linking point, mechanical properties

## Abstract

Protein-based hydrogels are considered ideal biomaterials due to their high biocompatibility, diverse structure, and their improved bioactivity and biodegradability. However, it remains challenging to mimic the native extracellular matrices that can dynamically respond to environmental stimuli. The combination of stimuli-responsive functionalities with engineered protein hydrogels has facilitated the development of new smart hydrogels with tunable biomechanics and biological properties that are triggered by cyto-compatible stimuli. This review summarizes the recent advancements of responsive hydrogels prepared from engineered proteins and integrated with physical, chemical or biological responsive moieties. We underscore the design principles and fabrication approaches of responsive protein hydrogels, and their biomedical applications in disease treatment, drug delivery, and tissue engineering are briefly discussed. Finally, the current challenges and future perspectives in this field are highlighted.

## 1. Introduction

**Figure 1:**
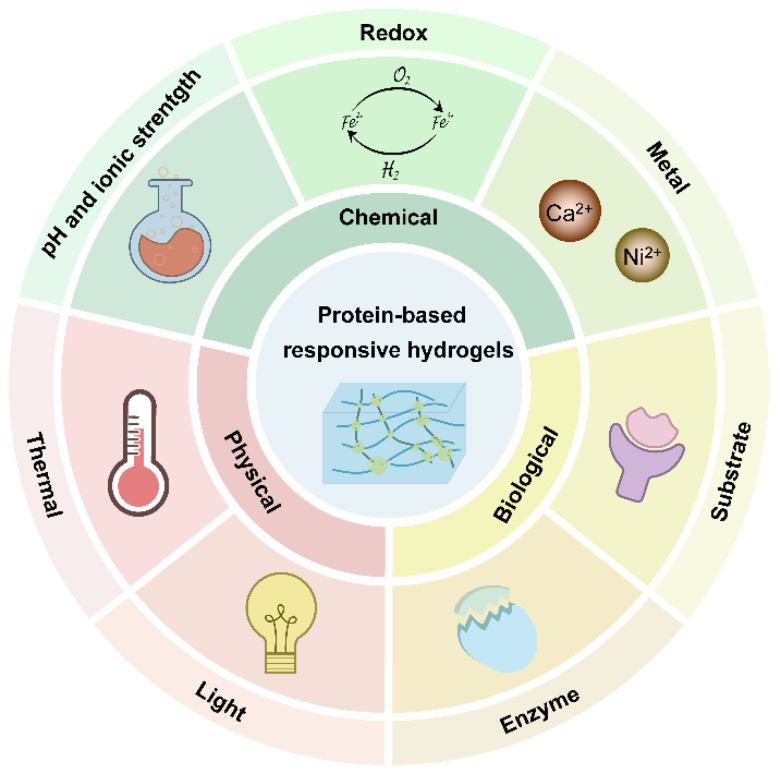
Overview of protein-based “smart” hydrogels that can respond to physical, chemical, and biological stimuli.

Hydrogels, attractive soft materials that mimic native extracellular metrics (ECM), have been extensively explored in tissue engineering, drug delivery, biosensors, and disease treatment [1,2,3]. In particular, hydrogels based on macrobiomolecules such as proteins have shown great promise for soft materials. As a crucial component of living systems, proteins play a diverse and essential role in various biological processes. They serve as catalysts for biochemical reactions, provide structural support, act as messengers for cell signaling and play an indispensable role in immune responses. These functions are vital for the proper functioning and maintenance of all living organisms. The intricate hierarchical organization of proteins, encompassing quaternary, tertiary, and secondary structures, confers them a diverse range of flexibility and mechanical properties, making them ideal building blocks for hydrogel fabrication [4]. Compared to chemical synthetic polymeric hydrogels, protein-based hydrogel exhibits a higher water content and a greater similarity to the natural extracellular matrix; their natural origins often result in lower cytotoxicity and superior biocompatibility. Furthermore, their ability to biodegrade naturally over time eliminates the need for invasive removal procedures, making them even more attractive for biomedical applications [5,6]. Although natural proteins such as collagen, gelatin, silk protein and globular proteins (e.g., bovine serum albumin (BSA), hemoglobin, etc.) have long been used as functional biomaterials, proteins extracted from natural sources poses certain drawbacks, including immunogenicity and quality variation from batch-to-batch [7]. By leveraging recombinant DNA technology, researchers can rationally engineer recombinant proteins with customizable sequences and functionalities [8]. The tailored motifs in proteins such as β-sheets, coiled coils and diverse reactive amino acids’ side chains can be harnessed for the design of protein-based responsive hydrogels, as these recombinant proteins exhibit a diverse range of structural variations in response to physical stimuli such as temperature, light or various chemicals and biomolecules (Figure 1) [9,10,11,12]. These microscopic alterations can be transmitted to affect the macroscopic properties of hydrogels, subsequently changing the behavior of the gel network, including the gel to solution (gel–sol) transition, causing swelling/de-swelling, and degradation and/or mechanical changes of hydrogel [9]. Via the integration of stimuli-responsive protein moieties with the newly developed bioconjugation methods, responsive protein hydrogels can be designed and fabricated using entirely genetically encoded proteins with tunable physical and mechanical properties or via hybridization with peptides or other synthetic polymeric networks to form a hybrid responsive protein hydrogel [13]. In this review, we have summarized the research’s progressions in the design, fabrication, and properties of responsive protein hydrogels. We narrated the key responsive units and cross-linking strategies that permit protein hydrogels with stimuli responsiveness. The potential applications of these responsive protein hydrogels in drug delivery, tissue engineering and other biomedical applications are briefly discussed. Finally, the current challenges and future perspectives are highlighted.

## 2. The Current Development of Responsive Protein Hydrogels

Until now, numerous proteins including elastin, collagen, BSA, fibrinogen, silkworm silk fibroin, amyloid fibers, and spider silk have displayed stimuli responses and have been applied for responsive protein hydrogels. However, the mechanical strength of natural protein-based hydrogels is weak, which limits its further biomedical applications. Special efforts have been made to improve these physical and mechanical properties by introducing permeant and reversible crosslinks which equip protein-based hydrogels with responsivity to external stimuli. The dynamic protein hydrogels can be grouped into three categories according to the type of external stimulus response, including physical, chemical, and biological responses. The represented protein hydrogels and the fabrication strategies, as well as their properties and applications, are comprehensively reviewed and briefly summarized in Table 1.

### 2.1. Physical-Responsive Hydrogel

#### 2.1.1. Photo-Responsive Hydrogel

Light-responsive protein hydrogel typically consists of polymeric building blocks harboring engineered photo-responsive proteins (photoreceptors) which can be categorized into reversible light sensing proteins such as Dronpa145N, UVR8 (UV-B resistance 8) and light-oxygen-voltage-sensing domain 2 (LOV2), and irreversible light responsive proteins such as C-terminal adenosylcobalamin (AdoB12) binding domain (CarHc) and photocleavable proteins (PhoCl). The widely used photo-switchable fluorescent protein Dronpa145N, developed by the Lin group, is a self-associated photo-responsive protein that maintains tetramers under 400 nm violet light and will depolymerize into monomers when exposed to 500 nm cyan light [59]. This process is triggered by the photo-induced trans-cis isomerization of the chromophore, which leads to the rearrangement of Arg66 and His193, which disrupts the tetrameric interface. Lyu et al. fused Dronpa145N with GB1 backbone proteins through SpyCatcher/tag chemistry to form a reversible protein hydrogel: (GB1-SpyCatcher)_3_-SpyTag-Dronpa145N. The rheology tests show the fast hydrogelation kinetics of the hydrogel and that the storage moduli G’ of the hydrogels increase from approximately 50 Pa (10% hydrogel) to approximately 1.2 kPa (20% hydrogel) in a protein concentration-dependent manner. Cyan light triggers the dissociation of the Dronpa145N tetramer and leads to the gel–sol transition, whereases violet light restores the Dronpa145N tetramer and resolidifies the hydrogel [19]. In another case, Dronpa145N was grafted with a four-armed Polyethylene glycol (PEG) using thiol-maleimide cross-linking to form a four-armed-PEG-Dronpa145N hydrogel. This hybrid hydrogel can transform into a viscous liquid under 505 nm light and then revert to a gel state after illumination at 405 nm [20]. Such hydrogels are capable of achieving rapid spatiotemporal control of the physical properties of hydrogel with potential applications for cell culture and drug release. Another photo-responsive protein, UVR8 from *Arabidopsis thaliana* undergoes a conformational swapping from dimer to monomer upon ultraviolet light irradiation [60]. Zhang et al. first utilized UVR8 as photo-responsive crosslinkers fused with a short peptide GGGWRESAI that could bind to the Tax-interacting protein-1 (TIP-1). TIP-1 was conjugated with Nap-GFFYGEK through a thiol-maleimide reaction, which further hybridized with photo-responsive group UVR8. The exposure to ultraviolet-B radiation results in the subsequent dissociation of the UVR8 homodimer and the dissolution of the hydrogel, consequently [18]. The photo-induced dimer–monomer switch between the dimeric and monomeric folding of UVR8-1 leads to a reversible gel–sol phase transition, presenting potential applications in photo-controlled drug/protein delivery and cell separation. Recently, a blue-light photoreceptor, LOV2, was developed as an optogenetic tool to remotely control protein activity and physiological processes. West et al. engineered a hybrid protein hydrogel containing LOV2 domains with PEG hydrogels and reversibly assembled ZDark1 (zdk1)-fused protein ligands onto the PEG-hydrogels under dark/light conditions [61]. Duan et al. exploit the hetero-dimeric interaction between LOV2 and its designed partner zdk1 to generate a light-responsive hydrogel named LOVTRAP which displayed shear-thinning and self-healing properties and served as an excellent injectable protein hydrogel. The interaction between LOV2 and zdk1 exhibits a higher binding affinity under dark light than that in blue light. They utilized a trifunctional elastomeric protein (GB1-SpyCatcher)_3_ as a polymer skeleton to generate (GB1-SpyCatcher)_3_-SpyTag-LOV2 and (GB1-SpyCatcher)_3_-SpyTag-zdk1. Blue light triggers the dissociation of LOV2 from zdk1. In the dark state, the LOVTRAP protein hydrogel formed a soft protein hydrogel with a storage modulus G’ of around 180 Pa and a loss modulus G” of around 40 Pa. Upon exposure to blue light, the hydrogel became even softer but did not melt, as evidenced by a rapid decrease in G’ to around 160 Pa and an increase in G” to approximately 60 Pa. This process was found to be reversible, with darkness reversing the process [15]. The bacterial adenosylcobalamin (AdoB12) binding domain (CarH) protein, a carotenoid synthesis transcriptional regulator, has been proven to respond to visible light through its C-terminal. CarHc tetramerizes when binding with AdoB12 in the dark and dissociates into monomers upon exposure to green (522 nm) or white light. Wang et al. constructed the elastin-like polypeptides (ELPs)-CarHc fusion protein using SpyCatcher/Tag chemistry. This CarHc-based hydrogel can dynamically undergo the photo-induced gel–sol transition, offering a strategy for the light-induced release/recovery of encapsulated protein molecules or mesenchymal stem cells (MSCs) [16].

**Figure 2:**
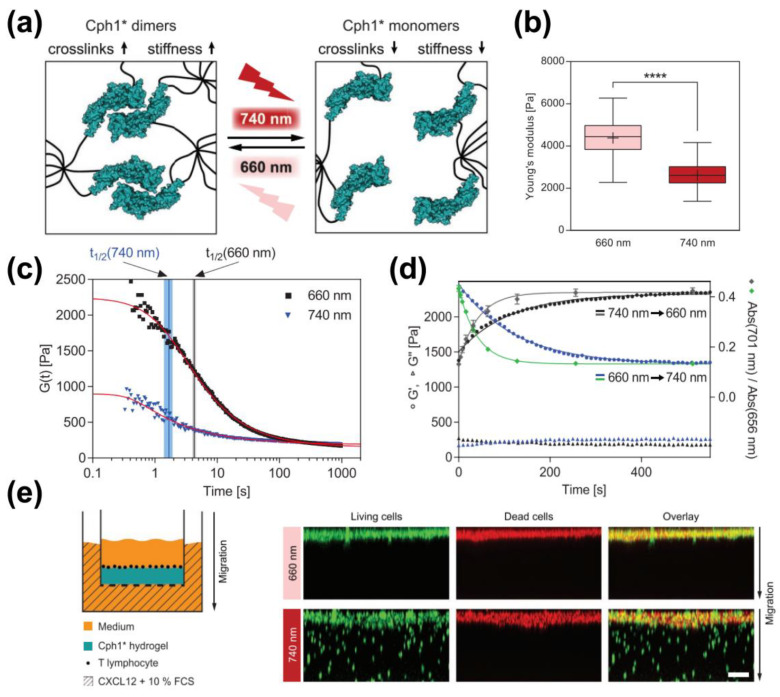
(**a**) The stiffness of the hydrogel is increased by the dimerization of Cph1* at 660 nm and decreased by the dissociation of Cph1* dimer at 740 nm. (**b**) Spatial distribution of the Young’s modulus of the surface of Cph1*-based hydrogels under 660 nm or 740 nm light exposure. (**c**) The relaxation modulus G(t) of gels irradiated with 660 or 740 nm light. (**d**) Kinetics of Cph1* photoconversion and G’ following optical switching between 660 and 740 nm light, respectively. (**e**) Cph1*-based hydrogels are employed in a transwell assay. Primary activated murine T lymphocytes were introduced onto the gel surface, followed by 8 h post-cultivation under 660 or 740 nm light before confocal imaging of the hydrogels. The white scale bar on the bottom right corner represents 100 µm. (This figure is reproduced with permission from ref. [9]. Copyright 2020, Wiley: Hoboken, NJ, USA).

Since UV or blue light exhibits low tissue penetration and toxic effects, red or far-red light may ameliorate the phototoxicity and enhance the penetration depth. The Weber group developed a phytochrome-based hydrogel by using the cyanobacterial photoreceptor (Cph1) (amino acid 1-514 with Y263F mutation) as a crosslinker (Figure 2). Cph1 Y263F (Cph1*) exists as a monomer at far-red light (≈740 nm) and forms a dimer under red light (≈660 nm). They fused Cph1* with RGD (Arg-Gly-Asp) and conjugated it with an eight-arm PEG through a cysteine-vinylsulfone (VS) addition. Red light (660 nm) triggers the dimerics of Cph1* and enhances the crosslink density of the PEG network, resulting in the stiffness of the hydrogel, as was indicated by a substantial drop in the average Young’s modulus when transitioning from 660 to 740 nm light. Far-red light (≈740 nm) caused Cph1* to shift to a monomeric state, leading to a reduction in the density of crosslinks and softening the hydrogel. When the 740 nm light source was turned off, the hydrogel underwent homogenous re-stiffening. This provided the possibility to manipulate the local mechanical environment, leading to investigations into the mechano-signaling pathways of human mesenchymal stem cells and the migration of T lymphocytes in response to changing mechanical environments (Figure 2) [9]. Using a similar strategy, The Weber group further incorporated the *Arabidopsis thalina*-derived phytochrome B (PhyB) and phytochrome-interacting factor 6 (PIF6) to develop an enhanced phytochrome-based hydrogel. The star-shaped PEG was coupled with PhyB and PIF6. Red light (600 nm) triggers PhyB and PIF6 to form a heterodimer and results in the formation of a hydrogel. Under far-red light (740 nm) exposure, the heterodimer dissociates and leads to a gel–sol transition [14]. Unlike Dronpa145N, UVR8, LOVTRAP, CarHc and Cph1, which undergo a light-induced transition of oligomerization, the photo-responsive protein PhoCl undergoes a light-triggered photo-cleavage. In darkness, it contains a C-terminal chromophore-bearing peptide within its β-barrel. Upon exposure to 400 nm light, the chromophore undergoes an irreversible β-elimination, leading to a cleaved peptide release from the β-barrel [62]. Shadish and his coworkers constructed a chimera of the PhoCl and PEG hydrogel network harboring the ligands that can be released in a dose-dependent manner upon blue light (400 nm) exposure [21]. Xiang et al. used PhoCl as a crosslinker to engineer two kinds of PhoCl-PEG hybridized hydrogels (Pho-Weak and Pho-Strong) with weakened and strengthened mechanical properties in response to light. Under 405 nm light, Pho-Weak was disrupted due to PhoCl cleavage. Meanwhile, the optimized cross-linking sites in the Pho-Strong-PEG hydrogel remained stable under PhoCl cleavage but induced active cysteines to outside exposure. The reaction between cysteine and the added maleimide strengthened the mechanical properties of the hydrogel. The mechanical properties of the engineered PhoCl-PEG hydrogel were adjusted by varying the density of thiol groups on the cysteines in the Pho-Weak-PEG or Pho-Strong-PEG protein. This resulted in a direct correlation between the amount of photo-cross-linking ligands on the protein and the overall strength of the hydrogel [22].

#### 2.1.2. Thermal-Responsive Hydrogel

**Figure 3:**
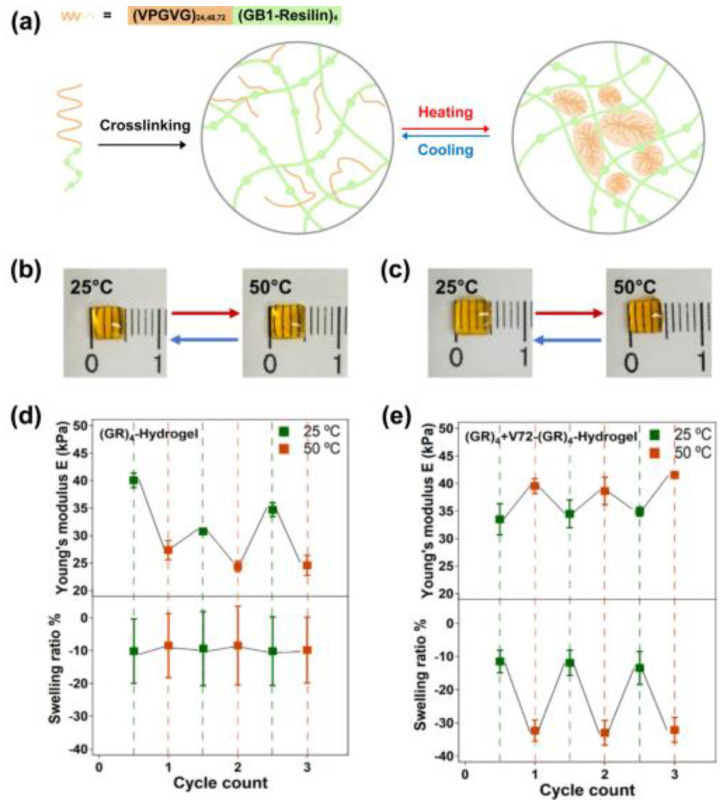
(**a**) Schematic illustration of Vn−(GR_4_) hydrogel preparation and temperature-induced phase transition. At room temperature, (GR)_4_ blocks bearing tyrosine in Vn−(GR_4_) are cross-linked via di−Tyr reaction and the non-tyrosine containing parts of Vn−(GR_4_) are dispositioned into the side chains. High temperature triggers aggregation of side chains, adding physical cross links and stiffening the hydrogel. (**b**,**c**) Size changes in (GR_4_) and Vn−(GR_4_) hydrogels at 25 °C and 50 °C. (**d**,**e**) Young’s modulus and swelling ratio of (GR_4_) and Vn−(GR_4_) hydrogels in three cycles of heating–cooling switch from 25 to 50 °C. (This figure is reproduced with permission from ref. [25]. Copyright 2020, ACS: Washington, DC, USA).

Thermal-sensitive hydrogels generally contain both hydrophobic and hydrophilic domains. Temperature changes determine the gelling states by affecting the swelling or causing the sol–gel phase transition. Thermo-responsive hydrogels can be categorized into lower critical solution temperature (LCST)-based or upper critical solution temperature (UCST)-typed hydrogels. LCST-based hydrogels are miscible at low temperatures and undergo gelation at temperatures high above the LCST, while UCST-based hydrogel forms a gel state below UCST and undergoes a gel to sol transition at the temperature above UCST. To date, the majority thermo-responsive protein hydrogels are constructed using ELPs) as the transition temperature can be tuned by chain length, concentration, or ionic strength. ELPs exhibit a hydrophilic nature with a loosely arranged structure below the LCST and transform to hydrophobic states with tightly packed structures above the critical temperature. Based on these features, Duan et al. selected VPGVG as the repeating unit of ELPs and integrated it with an elastomeric protein GB1-resilin (GR_4_) via a ruthenium-catalyzed photochemical reaction to form a recombinant protein hydrogel Vn-(GR)_4_, which undergoes a gel–sol transition when the temperature is below the critical temperature. Once heated above the critical temperature, the un-crosslinked ELPs within the hydrogel network will assemble and tighten the structure, reinforcing the mechanical properties of the hydrogel (Figure 3) [25]. Using a similar strategy, Parker et al. tethered VPGVG with the hydrophobic silk protein repeating unit GAGAGS through horseradish peroxidase-mediated tyrosine cross-linking. External heating induced the shrink of the hydrogel, enabling functional manipulation for tissue processing and histological analysis. SELP-based hydrogel can be also fabricated to form arrays in multi-well plates (such as 96-well plate) for the high-throughput histological analysis of organoids [24]. Dai et al. have successfully synthesized a hybrid protein-polymer hydrogel consisting of an ABC triblock. The hydrophobic A block is made up of a synthetic poly-trimethylene carbonate (PTMC), while thermosensitive ELPs are chosen for the BC blocks. At lower temperatures, the triblock forms micellar structures ranging in size from 10 to 60 nm. Upon heating above its Tt, larger particles with sizes of 200–300 nm are formed, indicating the formation of coacervates. As the solution becomes more concentrated, the viscosity gradually increases, ultimately leading to the formation of a hydrogel comprising a network of micron-sized particles. These hydrogels exhibit thermal reversibility with fast and distinct sol–gel transitions [13].

Compared to LCST-based hydrogel, less UCST-based hydrogel has been reported. Hill et al. rationally designed a UCST-type hydrogel using Q protein that could undergo a physically pentametric assembly between coiled–coiled nanofibers to form a porous network. A reversible thermo-responsive behavior was observed below or above the UCST point (16.2 °C) with 2.1% protein concentration. This characteristic grants it the ability to deliver drugs at physiological temperatures [10].

Gelatin, collagen, and ELPs exhibit thermo-responsive abilities. However, the weak hydrogen binding network results in poor mechanical properties. To overcome this limitation, chemical additives have been introduced to strengthen the hydrogen bonding network. Yang et al. introduced tannic acid to a gelatin-based hydrogel, enabling strengthened hydrogen networks between tannic acid molecules and gelatin chains. The resulting tannic acid-stablized gelatin hydrogel exhibited shape recovery properties in a temperature-dependent manner. This programmed shape change can be partially attributed to the hydrogen bond dissociation between gelatin and tannic acid upon elevated temperatures [23]. A similar design was performed using collagen and γ-glutamic acid (γ-PGA) hydrogels. Mixed collagen/γ-PGA forms a non-mobile gel at physiological temperatures, while breaking into turbidity and finally becoming a clear solution at high temperatures (above 42 °C). Based on this fact, the near-infrared (NIR)-responsive indocyanine green (ICG) has been applied to achieve a gel–sol transition with increased temperatures under NIR exposure. This approach harnesses the delivery of anticancer drugs like Doxorubicin (Dox) and offers potential feasibility for cancer thermotherapy [27].

Most thermotensile hydrogels undergo temperature-induced phase separation below or above LCST and UCST. However, very few thermal-sensitive hydrogels can perform the gel–sol–gel transition. The Xia group pioneered the development of a dual thermosensitive hydrogel by using the carboxyl-terminal domains (CTD) of spider dragline silk protein. The C-terminal domain consists of five α-helix bundles. The hydrogel was formed at a low temperature due to the strong hydrogen bonds and hydrophobic interactions between the globular structures of the folded proteins. With the temperature increases, the hydrogen networks disintegrated gradually and exhibited a gel–sol transition. At a high temperature, the hydrophobic residues previously concealed within proteins were exposed and crosslinked with each other, forming an irreversible hydrogel [28]. They further combined the ELP block with the C-terminal domain of spider major ampullate spidroin 1 (CTD) to construct a chimeric dual-thermosensitive hydrogel. The CTD exhibits UCST behavior and aggregates at lower temperatures while the ELP block maintains LCST behavior and aggregate at higher temperatures. Below the critical CTD temperature (Tt), the ELP-CTD forms a stiff hydrogel network. At room temperature, that between the two Tts of CTD and ELP, the hydrogel was melted into a solution. This gel to sol feature can be used for cell storage and release. Above the Tt of ELP (37 °C), ELP-CTD adopts a soft hydrogel which exerts shear-thinning properties for injection [26].

### 2.2. Chemical-Responsive Hydrogel

#### 2.2.1. Metal Ion-Responsive Hydrogel

**Figure 4:**
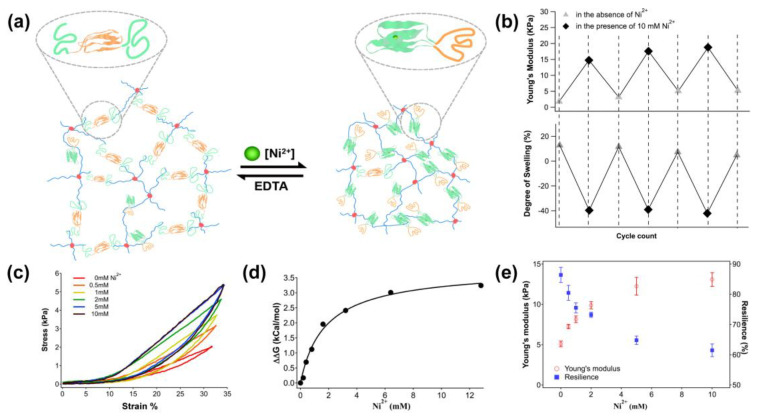
(**a**) Schematic illustration of the conformation changes of His-tagged MEP in the Ni^2+^-bound or free state. (**b**) Young’s modulus and swelling ratio of GR−(G−MEPHH−R)_2_ hydrogel in three cycles of switching between Ni^2+^-bound or free state. (**c**) Stress–strain curve under Ni^2+^ titration. (**d**) The thermodynamic stability of GL5HH−I127 improved with higher concentrations of chelated Ni^2+^. (**e**) The Young’s modulus and resilience of the hydrogel under different concentrations of Ni^2+^. (This figure is reprinted with permission from ref. [31]. Copyright 2016 ACS: Washington, DC, USA).

Metal binding proteins are ubiquitous in living cells and the metal–protein interactions can serve as a reversible cross-linking approach for manipulating the mechanical properties of protein hydrogels [29]. Metal-histidine coordination is prevalent in many metalloproteins and has enlightened the development of metal-histidine-based peptide and protein hydrogels. Wang et al. designed a CEC-D protein hydrogel. The C domain is a coiled-coil domain sequence from a cartilage oligomeric matrix that can bind with a small molecule, and the E domain is an elastin-like polypeptide that can be responsive to divalent metal ions. The coordination of histidine in CEC-D with Cu^2+^ and Zn^2+^ can increase the stiffness and viscoelasticity of the hydrogel, while Ni^2+^ triggers the opposite effect. Thus, the CEC-D protein hydrogel can be used in a tunable drug release by adding different metal cations [30]. Sun Group utilized the distinguished affinity between histidine-Co^3+^ and -Co^2+^ coordination to fabricate a protein hydrogel [32]. Jiang et al. harnessed the specific coordination interactions between the metal (Ni^2+^, Zn^2+^, Co^2+^) and His-tag to assemble His6-tagged-CarHc proteins into a photo-responsive hydrogel [33]. Kong et al. developed a metal-chelation protein hydrogel by introducing His-tag into s mutually exclusive protein (MEP) to create protein hydrogels named GL5HH-I27 (Figure 4). The chelation of His-tag and Ni^2+^ triggered distance changes between the cross-linking points in the gel, resulting in an equilibrium shift between the two mutually exclusive conformations, and consequently affecting the cross-linking density of the hydrogel [31].

A series of calcium-responsive protein hydrogels have been reported based on the calcium-facilitated calmodulin (CaM) hetero-oligomerization. CaM can bind more than 300 different target proteins in a Ca^2+^-dependent manner [63]. Gallivan et al. utilized Ca^2+^-induced cross-linking between CaM and the calmodulin binding domain (CBD) to form a CaM-containing protein hydrogel. Such hydrogel displays a reversible property in the presence and absence of ethylenediaminetetraacetic acid (EDTA) [36]. Luo et al. developed ELP-based Ca^2+^-responsive hydrogels by covalently assembling ELPs with calmodulin (CaM) and M13-peptide using SpyCatcher/Tag chemistry. The addition of calcium induces the binding of CaM and M13, which increases the stiffness of the hydrogel. These calcium-responsive hydrogels can be applied for 3D cell culturing [35]. Overall, the metal (nickel, zinc, or calcium)-based gelation provides a general approach to facilitating the reversible interchain-cross-linking between protein building blocks.

#### 2.2.2. Redox-Responsive Hydrogel

The redox potential of cysteine plays a crucial role in protein structure and function. Reversible thiol-disulfide conversions facilitate the preparation of protein hydrogel with redox responsivity. Many cysteine-rich proteins, such as BSA [64], silk elastin-like peptides (SELPs) [37], collagen-like proteins (CLPs) [43] and keratin [40] have been transformed to redox-responsive hydrogels, making them promising candidates for various biomedical applications, including drug delivery and tissue engineering. Nikfarjam et al. prepared a BSA-based hydrogel. The use of the chemical denaturant guanidine hydrochloride (Gdm-HCl) can unfold the BSA and increase the hydrophobic interactions of buried cysteines which were exposed and cross-linked through disulfide bond formations in the presence of hydrogen peroxide. The unfolding of BSA induced by the denaturant enabled an increase in the cross-linking density and the stiffness of the hydrogel [38]. The Xia group reported a recombinant silk-elastin-like protein hydrogel (SELPs) in which cysteine residues are introduced into the elastin block with varying silk-to-elastin ratios. Hydrogen peroxide (0.05%) triggered the formation of the disulfide-bonded cross-linked SELP hydrogel and dissociated under a reductive environment [37]. As a result, the redox sensitivity of engineered cysteine residues, combined with the thermal responsive properties of elastin, endowed SELP hydrogels with dual responsiveness, providing potential applications in drug delivery and tissue engineering. Jia et al. endeavored to construct a recombinant protein hydrogel consisting of collagen-like proteins (CLPs), RGD domains and cysteine-proline-proline-cysteine (CPPC) peptides. One or two paired cysteines were introduced at both the N-and C-terminals of the protein and cross-linked one another under oxidative conditions to form a hydrogel network. It can sense reduced environments and subsequently lead to a gel–sol transition. This strategy offers a new biocompatible way of developing advanced tunable biomaterials that can be used for wound healing and mitigate oxidative stress injury in diabetes [43]. Asai et al. constructed mutated ELPs with cysteine residues in the elastin block, which enable chemical cross-linking through disulfide bond formation. They found that the silk-to-elastin block ratio at 1:4 displayed redox-sensitive properties in the solution [44]. The disulfide cross-linking could also be adapted with mutually exclusive proteins (MEP) in which two cysteines are introduced to the MEP domain. The Li group developed a series of redox-responsive protein hydrogels based on MEPs. In the GB1-R-(G-MEP-R)_2_ system, the disulfide bond formation affects significant conformational changes as the contour length reduces almost ten-fold from reducing to oxidizing conditions. In another folding/unfolding system, GL5- (a mutant of GB1) and I27-based hydrogels were engineered by introducing two cysteines into GL5 to form a GL5CC-I27-based hydrogel. In the oxidated state, the host domain GL5CC is folded while the guest domain I27 is unfolded. In the reduced state, the host domain GL5CC is unfolded while the guest domain I27 remains folded, which leads to an increased swelling ratio in the hydrogel [29]. Thus, this hydrogel can be applied in microfluidics and drug delivery systems in the reduced state. Subsequently, they split the GL5 domain into fragments, G_N_ and G_C_, and performed a self-assembly under reductive conditions at 37 °C. The reconstituted G_N_/G_C_ assembly can be further stabilized through intermolecular disulfide bonds formed under oxidative conditions at 4 °C [45].

**Figure 5:**
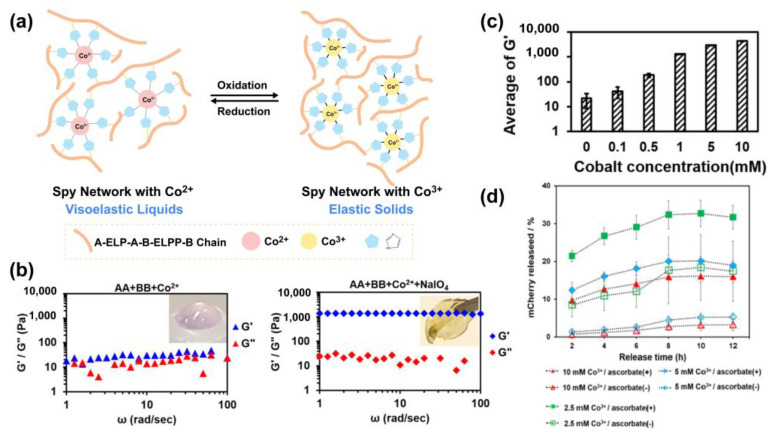
(**a**) Assembly of cobalt coordination protein into hydrogels. SpyTag−ELP−RGD−ELP−SpyTag (AA) and His6-SpyCatcher−ELP−RGD−ELP−SpyCatcher (BB) chelate with Co^2+^. Oxidation of Co^2+^ to Co^3+^ triggers Spy network transition from a viscoelastic liquid to a highly elastic solid. (**b**) Dynamic frequency sweep tests on AA + BB + Co^2+^ and AA + BB + Co^3+^. The low storage modulus G’ of Co^2+^ complex indicated that the His−tag−Co^2+^ coordination was less stable than His−tag−Co^3+^ coordination. (**c**) The Young’s modulus of the hydrogel in different cobalt concentrations. Higher cobalt concentration can increase the stiffness of the hydrogel. (**d**) This hydrogel can release the His6-tagged mCherry upon the reduction of Co^3+^ to Co^2+^ within ascorbate solution. (This figure is reprinted with permission from ref. [32]. Copyright 2019 ACS: Washington, DC, USA).

Apart from the internal disulfide cross-linking within proteins, Renay et al. developed a cross-linking strategy by introducing a small redox-responsive crosslinker 3,3′-dithiobis (sulfosuccinimidyl propionate) (DTSSP) to form a recombinant resilin-like hydrogel, RZ10-GRD, through a succinimide-lysine crosslink [65]. The intramolecular disulfide bond in DTSSP was cleaved in a reducing environment and further resulted in the rapid degradation of hydrogel networks. This degradable redox-responsive material is promising for tissue engineering and targeted drug delivery. Cysteine enriched keratin proteins have been manufactured to construct responsive hydrogels based on a disulfide strategy. Cao et al. developed a disulfide shuffling strategy by converting intramolecular disulfide bonds to intermolecular disulfide bonds [40]. The intramolecular disulfide bonds in native keratin were first reduced to cysteine in response to a high level of reduced glutathione (GSH) and could be further oxidized to form intermolecular disulfide bonds. Such keratin hydrogel has a denser disulfide cross-linked structure that exhibits a higher mechanical strength and redox-responsive capacity, providing a potential capability for tissue engineering and drug release. Sun Group leveraged the His-tag cobalt coordination and the Co^2+^/Co^3+^ ligand dynamic exchange to develop a recombinant protein hydrogel. Because Co^2+^-His6-tagged complexes are thermodynamically less stable than those of Co^3+^-His6-tapgged complexes, the properties of the hydrogel were transformed from being a viscoelastic liquid to a highly elastic solid upon the oxidation of Co^2+^ to Co^3+^. This cobalt-based hydrogel is suitable to modulate cell–substrate interactions, offering a new strategy for protein delivery and cell growth/proliferation (Figure 5) [32].

#### 2.2.3. pH- and Ionic Strength-Responsive Hydrogel

Protein hydrogels can respond to solution pH and ionic strength by undergoing structural and property changes such as surface activity, chain conformation, solubility, and configuration. Some protein hydrogels also exhibit different properties, such as degradability, depending on the pH and ionic strength of their chemical bonds and van der Waals force [66]. The pH was found to have a significant impact on the formation of BSA hydrogel. The composition of α-helix decreased with lower pH, which is in good agreement with the gel–sol conversion of BSA hydrogel in the different pH levels. In near-neutral pH, BSA adopts a globular heart-shaped structure (N-isoform) and transforms into a partially elongated cigar-shaped structure (F-isoform) at pH lower than 4.3, and further denatures into a completely expanded structure (E-isoform) under extremely acidic conditions (pH lower than 2.7) [67]. The pH of bacteria-infected wounds is around 5.5, lower than the normal pH of the human body. So, pH-responsive protein hydrogel is promising for wound healing [68,69]. Perovic et al. used a recombinant oyster shell nacre protein (r-n16.3) which possesses intrinsic disorder, aggregation regions and a nearly equivalent number of anionic and cationic side chains [70] to create a pH and ionic strength-responsive hydrogel [50]. When pH is lower than the pI (4.85) of r-n16.3, the protonation of carboxylate residues in r-n16.3 induces charge shielding and alters the assembly of r-n16.3, resulting in a decrease in the particle diameters of the r-n16.3 hydrogel and an increase in the protein film formation on the mica surface. After the addition of NaCl, the charge of anionic and cationic residues can be partly induced by Na^+^ and Cl^−^ and lead to smaller particles being clustered into large hydrogel particles. The addition of Ca^2+^ can also lead to a similar clustering effect, even in the formation of linear or fiber-like chains. This may because Ca^2+^ can mediate salt-bridging interactions between the side chains of proteins. He et al. fabricated a gelatin-based hydrogel that can undergo quick degradation in response to CO_3_^2−^, Cl^−^ and I^−^ ions, providing potential applications for tissue reconstruction [71]. The Hofmeister series is a class of ions influencing protein stability in solution, which can stabilize proteins in the presence of SO_4_^2−^, HPO_4_^2−^, Cl^−^, Br^−^, NO_3_^−^, ClO_3_^−^, I^−^, ClO_4_^−^, SCN^−^. Jiang et al. prepared ion-responsive regenerated silk fibroin/gelatin (RSF/G) hydrogels using horse radish peroxidase (HRP) and H_2_O_2_ to cross-link the RSF and further immersed it in (NH_4_)_2_SO_4_ solution to cross-link physically and toughen the hydrogel. In contrast, the hydrogel immersed in solution containing CO_3_^2−^, I^−^ or Cl^−^ quickly degraded in PBS [72].

A small number of chemical groups can exhibit accelerated degradation or hydrolysis behavior in acidic or alkaline environments. By taking advantage of the rapid hydrolysis characteristics of these chemical bonds under the stimulation of acids and alkalis, a series of pH-responsive degradation hydrogels have been prepared by introducing acid- and alkaline-stimulated hydrolysable chemical bonds (imine [73,74], oxime [75]) into protein hydrogels. The pH-sensitivity of the reversible Schiff base-linkage offers a platform on which to fabricate dynamic biomedical hydrogels. Yuan et al. adopted the cross-linking of glutaraldehyde (GA) with diphenylalanine (FF) and proteins to confer self-healing hydrogels. Various proteins, including hemoglobin (Hb), myoglobin (Mb), BSA, human serum albumin (HSA), and fibrinogen (FIB) can be cross-linked with FF and GA to assemble FF-dipeptide-protein hydrogels. These self-immobilized dipeptide-protein hydrogels may undergo a gel–sol transition when exposed to acidic or basic pH stimuli [47]. Fu et al. developed a BSA-based self-healing hydrogel material based on the reversible Schiff base formation. They used glutaraldehyde to cross-link BSA hydrogel with glucose oxidase (GOX) and catalase (CAT). In this system, the feature of the reversible imine covalent attachment of the glutaraldehyde to the lysine residues of BSA, GOX and CAT proteins imparts to the protein hydrogel a self-healing function. In this process, GOX assists in the oxidation and hydrolysis of glucose to gluconic acid which decreases the pH of the protein hydrogel. The pH reduction promotes the breaking of the Schiff base to release lysine residues in BSA, GOX and CAT proteins [46]. Tumor cells exhibit a preference for glycolysis which leads to the increased fermentation of glucose to lactate. The subsequent accumulation of lactate lowers the pH of the tumor’s microenvironment. This metabolic characteristic suggests the potential for utilizing pH- and ionic strength-responsive protein hydrogels in cancer treatment.

**Figure 6:**
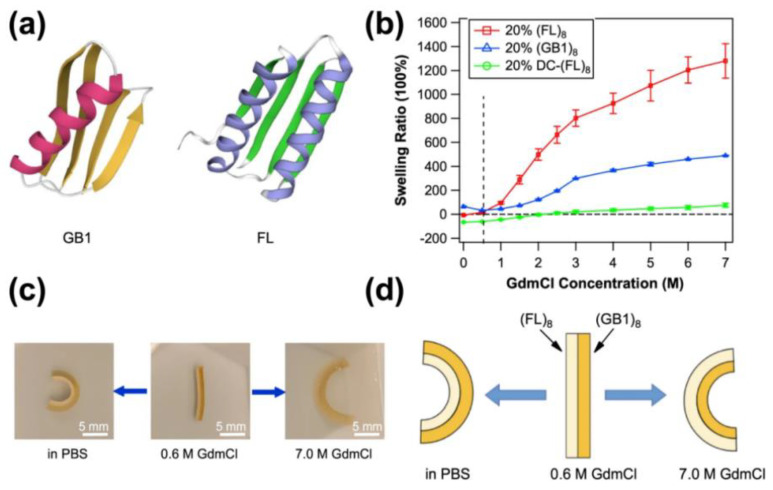
(**a**) The 3D structure of folded globular GB1 domain (left) and FL domain (right). (**b**) The swelling ratios (SR) of hydrogels at different GdmCl concentrations. (**c**,**d**) The bidirectional bending behaviors of (GB1)_8_/(FL)_8_ bilayer hydrogel. The bilayer strip exhibited a curvature towards the (FL)_8_ side in PBS and curved towards (GB1)_8_ side when the concentration of GdmCl exceeded 0.6 M. (This figure is reproduced with permission from ref. [48]. Copyright 2022, Springer: Berlin/Heidelberg, Germany).

Chemical denaturants can denature proteins and significantly reduce the mechanical strength of the hydrogels. Several globular proteins can fold and unfold reversibly in response to denaturants. Li Group engineered a bilayer-shape memory hydrogel based on the folding-unfolding mechanism and distinct denaturant-dependent swelling properties of two elastomeric proteins, GB1 (B1 immunoglobulin-binding domain of protein G of streptococcal) and the ferredoxin-like protein FL (a computationally redesigned variant of the designer protein DiI_5). They fused eight tandem repeats of GB1 and FL to form a (GB1)_8_-(FL)_8_ hydrogel [76]. Due to the distinct swelling behaviors of GB1 and FL in response to the denaturant, the denaturant GdmCl induced the asymmetric unfolding of (GB1)_8_ and (FL)_8_. In a PBS buffer, the (FL)_8_ layer contracted while the (GB1)_8_ layer expanded, causing the hydrogel to bend towards the (FL)_8_-layer side. In high concentrations of GdmCl, the (FL)_8_ exhibited a significantly higher swelling ratio compared to the (GB1)_8_ layer. The difference in swelling degrees led the hydrogel to bend towards the (GB1)_8_-layer side. As a result, bidirectional bending behavior could be observed in different solution environments. This work provides new insights for the design and production of innovative shape-morphing materials (Figure 6) [48]. Khoury et al. developed another shape memory material using BSA grafted with polyelectrolytes such as polyethyleneimine (PEI) and poly-L-lysine (PLL). PEI improved the stress-sustaining limits of the BSA hydrogel and induced the stiffness of the BSA hydrogel. Thus, the PEI-BSA hydrogel undergoes reversible softening and stiffening between the circle of native (TRIS buffer) and denaturing (GuHCl 6 M) conditions [49].

### 2.3. Biologically Responsive Hydrogel

#### 2.3.1. Ligand-Responsive Protein Hydrogel

The conformational change of the protein-ligand recognition has been employed for the construction of the responsive protein hydrogel. The ligand-receptor binding of maltose and maltose-binding protein (MBP) was used as an ideal model to investigate the relationship between individual protein–ligand interactions and the macroscopic properties of a cross-linked MBP. Because the mechanical and thermal stability of MBP protein increased with the addition of maltose, Hughes et al. engineered a maltose-bound protein (MBP) hydrogel by transforming the stability of the building block into a whole cross-linked hydrogel network. The mechanical strength of MBP hydrogels was increased in the presence of maltose. This ability to control the mesoscopic stability of the protein building blocks provides an opportunity to exploit dynamically responsive hydrogels due to protein–ligand interactions [58]. They further investigated the thermodynamic stability of unbound MBP (U-MBP) and the more stable maltose-bound MBP (MB-MBP) via an increased urea treatment. The thermodynamics of the MBP building blocks can be controlled by tuning the concentration of urea in the hydrogels, which in turn affects the hydrogel’s swelling behavior, resulting in changes in the stiffness of protein hydrogel networks [12].

**Figure 7:**
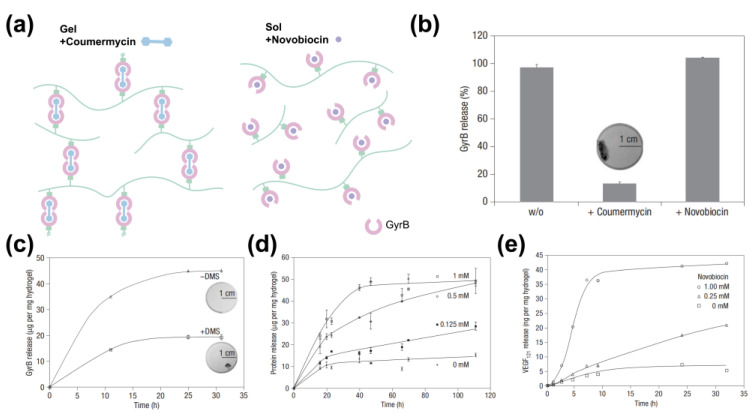
(**a**) GyrB grafted to polyacrylamide was dimerized by the addition of the antibiotic coumermycin, resulting in hydrogel formation (left). Addition of increasing concentrations of novobiocin dissociated the GyrB subunits, leading to dissolution of the hydrogel (right). (**b**) The GyrB- released ratio of hydrogel incubated separately in the presence of coumermycin, novobiocin and the lack of any antibiotics. Photograph of the hydrogel is illustrated as well. (**c**) The hydrogel was formed with coumermycin−dimerized GyrB and further cross-linked with (hollow triangle) or without (hollow square) dimethyl suberimidate (DMS). After swelling in PBS overnight, the hydrogel was treated with 1 mM novobiocin and the release of GyrB was measured. Photographs of the hydrogel are illustrated as well. (**d**) GyrB release curve of hydrogel incubated with different concentrations of novobiocin. The degree of hydrogel disintegration was measured by quantification of GyrB released. (**e**) VEGF_121_-release curve of hydrogel under different concentrations of novobiocin. The protein−released kinetics were adjustable due to the hydrogel characteristics. (This figure is reproduced with permission from ref. [56]. Copyright 2008, Springer: Berlin/Heidelberg, Germany).

Owing to the conformational shift of calmodulin (CaM) mutants upon the binding of ligands such as phenothiazine or trifluowere, in the early example, engineered calmodulin (CaM) containing two SH groups was grafted with PEG_575_-diacrylate through a Michael-type addition reaction. The binding of ligand trifluoperazine with CaM induced a conformational shift which in turn triggered the hydrogel volume changes [57]. Sui et al. cross-linked CaM with four-arm PEGs to form a hybrid hydrogel. The CaM protein domain adopts a relaxed conformation in the presence of Ca^2+^ while switching to a tensed conformation after binding with ligands (phenothiazine or trifluoperazine), resulting in the contraction of the CaM-PEG hydrogel. The addition of ethylene glycol tetraacetic acid (EGTA) can chelate the Ca^2+^ in the system, resuming the swelling of the hydrogel [77]. Yuan et al. utilized the adenylate kinase (AKe) protein mutant (AKtm, C77S, A55C, V169C) as a crosslinker conjugated with synthetic polymers N-2-hydroxypropylmethacrylamide (HPMA) to form a hydrogel through the thioene reaction of Ake’s two cysteine residues (Cys 169 and Cys 55). The interactions of ATP induced a conformational transition of AKtm from an open to a closed state which decreased the distance between the two cysteine residues. As a result, the Ake-based hydrogel de-swelled in response to an elevated ATP concentration [55]. The competitive binding of ligands to protein antibiotics to receptors can also be a source of hydrogel responsiveness. Genetically engineered is the receptor for the antibiotics coumermycin and novobiocin. The binding stoichiometry of bacterial gyrase subunit B (GyrB) to coumermycin is 2:1 and 1:1 for novobiocin. Ehrbar et al. engineered a GyrB-based hybrid hydrogel using GyrB and polyacrylamide. The coumermycin-stablized GyrB dimer could be quickly disrupted to a GyrB monomer by adding a higher amount of the antibiotic novobiocin which can compete with the binding of GyrB and coumermycin. The transition of the GyrB dimer to a monomer resulted in a gel–sol transition which could be used for drug release. Using this system, a protein drug, human vascular endothelial growth factor (hVEGF), can be released in response to novobiocin concentrations. A single oral dose of novobiocin can gradually release effective hVEGF in human plasma, indicating great promise for the controllable drug release *in vivo* (Figure 7) [56].

#### 2.3.2. Enzyme-Responsive Protein Hydrogel

The cleavage of proteins/peptides at the specific recognition sites of enzymes can be applied to build enzyme-stimulated protein hydrogels. The resulting hydrogels can be dissociated in the presence of a specific cleavage enzyme, holding great potential for drug delivery in the human body where the enzyme could activate the hydrogel to release the drug at the target site. The currently reported enzyme-responsive protein hydrogels are mainly based on enzyme-catalyzed crosslink degradation. The feasibility of Matrix metalloproteinase 7 (MMP7) to cleave peptides is utilized to develop enzyme-responsive protein hydrogels. MMP7-cleavable peptides were tethered with the streptococcal collagen-like 2 (cl2) scaffold protein to form a hydrogel that can exhibit a swelling ratio change in response to MMP7-catalyzed peptide cleavage. Since MMP7 is secreted by human mesenchymal stem/stromal cells (hMSCs) during chondrogenesis, the hMSCs encapsulated in this collagen-mimetic hydrogel can be released via cellular MMP7 to facilitate cartilage regeneration [51,52,53]. Ji et al. applied glutaraldehyde to link polyallylamine hydrochloride (PAH) and a recombinant protein TP that consisted of a specific thrombin-cleavable site. The resulting hybrid protein-polymer hydrogel exhibited a high swelling ratio of 900% when 15% TP was cross-linked with PAH. The presence of thrombin enables a controlled hydrogel decomposition at a specific time point, making this PAH-composite hydrogel suitable for use in time-controlled release applications [54].

## 3. General Principles for the Design and Fabrication of Responsive-Protein Hydrogels

**Figure 8:**
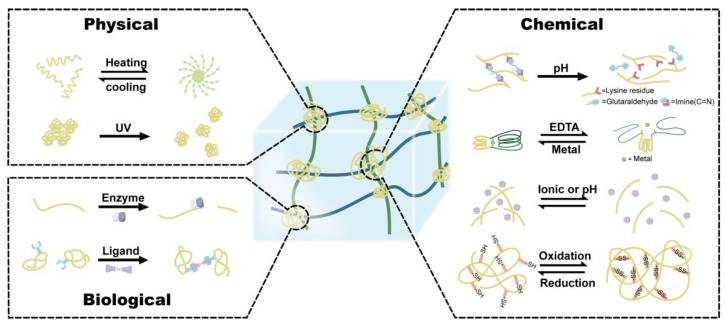
General fabrication strategies to construct responsive protein-based hydrogels. Protein-based or related responsive units were served as reversible crosslinkers that could be incorporated into the hydrogel to exhibit tunable mechanical, physical, and biochemical properties, providing controllable responsiveness towards environmental cues, such as temperature, light, ionic strength, redox stress and metals, as well as biological enzymes and ligands.

Creating responsive hydrogels involves the intentional design of building blocks containing responsive domains. The characterization of a protein hydrogel network is influenced by several key parameters such as the size of the protein, the fraction of protein in the swollen state, and the density and distance of crosslinks. These parameters are interconnected and the manipulation of the cross-linking density or distance can effectively regulate mechanical stiffness, swelling, and mesh size. Therefore, the introductions or adjustments of crosslinks in protein hydrogels are crucial in determining the physical and mechanical properties. Intrinsic physical crosslinks, such as hydrogen bonding, hydrophobic interactions and electrostatic interactions within the protein result in dynamic, rapid, but unstable hydrogel networks [23,49]. Therefore, additional chemical crosslinks can be introduced to stabilize the gel network. To date, numerous efficient cross-linking methods are constantly being developed to introduce covalent crosslinks in protein hydrogels. Among them, genetically encodable cross-linking chemistry has been implemented to establish intermolecular irreversible covalent bonds in protein hydrogels, such as SpyCatcher/Tag chemistry [78], ruthenium-mediated di- or tri-tyrosine cross-linking and cysteine-involved bisulfide cross-linking [45]. Additionally, enzyme-catalyzed cross-linking approaches such as sortase A [79,80,81] and split intein [82] have also been implemented for the ligation of shorter peptides into covalently cross-linked networks. These permanent cross-linking methods establish new static networks that empower the hydrogels with stable mechanical and physical properties. Apart from permanent cross-linking, reversible cross-linking involving the physical, non-covalent or cleavable covalent bonds are pivotal in shaping the dynamic characters of protein-based hydrogels. Proteins can either serve as reversible/cleavable crosslinkers integrated with load-bearing modules (such as proteins, peptides, or other polymeric networks) or as building blocks conjugated with small reversible/cleavable chemical crosslinkers. External stimuli such as temperature, light, pH, redox stress, or ligands can trigger conformational changes or folded structures’ reassembly of crosslinkers, altering the density or length of reversible cross-linking networks (Table 1). In this section, we summarized the latest strategies for tuning or introducing static and/or reversible cross-linking points to convey the hydrogel’s responsive properties (Figure 8).

### 3.1. Photo-Responsive Crosslinks

Amongst the environmental stimuli, light has been widely used for manipulating and monitoring biological functions in cells and tissues, owing to its non-contact and controllable regulation in a spatial and/or temporal manner. Biological photoreceptors display biocompatible photo-responsiveness in physiological environments, making them ideal crosslinkers in photo-responsive protein hydrogels whose physical and mechanical properties were dynamically regulated by light. The current photo-responsive protein hydrogels can be classified into three families due to different responsive mechanisms: (1) photo-induced oligomerization transition—Dronpa145N [83], UVR8 [60], Cph1 [9] and CarHc [16,17] can undergo homo-oligomerization under dark conditions and dissociate in response to light irradiation, while PhyB and PIF6 can form heterodimers under far-red light (740 nm) and dissociate under red light (660 nm) [14]; (2) photo-induced conformational change—in the LOV2-Jα system, blue light triggers the Jα-helix to move away from the LOV2 core, causing changes in the length of crosslinkers which in turn affects the mechanical properties of the hydrogel [15,84]; and (3) photo-induced cleavage proteins such as PhoCl that can initiate the cleavage of their cognitive sequences under light irradiation. By controlling the intensity, wavelength, and exposure time of the light irradiation, the properties of the hydrogel including its mechanical strength, degradation rate, and swelling behavior can be finely tuned [21,22].

### 3.2. Temperature-Induced Reversible Phase Transition

Proteins can undergo phase transitions in response to solution environments such as temperature, pH, and pressure. Phase transition can alter the cross-linking density and can be utilized to fine-tune the properties of hydrogels. ELPs derived from the hydrophobic domain of elastin protein represent the most well-known thermal-responsive protein hydrogels, exhibiting a lower critical solution temperature (LCST) behavior. The repetitive VPGXG (X represents any amino acid except for proline) sequences in ELPs can undergo reversible phase transitions at different temperatures. Below the transition temperature (Tt), ELPs remain in hydrophilic states with high solublity properties. Above the Tt, ELPs become hydrophobic and aggerate in solution due to conformational changes. The temperature-induced phase transition enables ELPs or ELP-containing protein hydrogels to form hierarchical structures including micelles, vesicles and aggregates. Gelatin and Protein Q display a UCST-type behavior which can form random single coils in an aqueous solution above a UCST of 40 °C and can transform into triple helical structures upon cooling [10,85]. Unlike ELPs only displaying a LCST, the C-terminal domain of spider silk (CTD) exhibits dual thermal-sensitive effects. It gelates around physiological temperatures and transforms to micelles at lower temperatures, while aggregating at high temperatures [28].

### 3.3. Reversible Covalent Crosslinks

Proteins consisting of cross-linkable amino acids that contain reactive thiols or amine groups can undergo covalent cross-linking though oxidation or through reacting with small molecules such as glutaraldehyde. The disulfide bond formation via the oxidation of two adjacent thiol groups in cysteine residues has been employed for protein hydrogel preparation. Moreover, the disulfide bonds can also be reduced to cysteine under reductive environments, leading the reversible thiol-disulfide conversion which further affects changes in protein hydrogel cross-linking density. The inherent features of cysteine-rich proteins, such as BSA [64], keratin [40] and resilin [11] have been widely used for the preparation of redox-responsive hydrogels. Moreover, the ability to engineer a protein sequence with specific cysteine residues using recombinant protein technology permits the precise control of redox-responsive moieties which can alter the physical and mechanical properties of protein hydrogels. This feature can be exploited for practical applications such as drug release in reductive environments [37]. Like cysteine-mediated disulfide cross-linking, the formation of Schiff bases formation can also achieve reversible reactions under physiological conditions, which are typically stable when pH is above 7.4, and hydrolyze to aldehyde and amine when pH is below 5 [86]. This pH-mediated disintegration of Schiff bases in protein hydrogels can trigger a gel–sol transition [87,88]. The principles for designing protein hydrogels using Schiff bases’ crosslinks are reviewed in detail by Sahajpl et al. [89].

### 3.4. Reversible Non-Covalent Crosslinks

Unlike covalent bonds introduced in permanent cross-linkages, proteins exhibit versatile non-covalent binding with ligands such as metal ions, small biomolecules and antigens, etc. Amino acid residues such as histidine, cysteine, and asparagine can interact with metal ions (e.g., Ca^2+^, Zn^2+^ and Ni^2+^ et al.) to form soluble metal coordinate complexes which can be reversed upon the addition of EDTA [36]. This metal–protein chelation triggers the reversible conformational protein changes that can be harnessed to engineer protein-based hydrogels with tunable mechanical properties [29,32]. In addition to metal ions, the inherent feature of proteins to selectively bind with ligands can be implemented to develop responsive hydrogels. The pioneering work conducted by Murphy and co-workers introduced a dynamic protein, calmodulin (CaM), as a partial crosslinker in hydrogel networks, with the addition of the small CaM-binding drug trifluoperazine resulting in a dramatic conformational change of CaM, leading to significant volume changes in the protein hydrogel [77]. Following these ligand-binding-induced folding properties, CarHc [16,17], adenylate kinase [55] and GyrB [56] have been employed for generating responsive protein hydrogels as tunable ligand-responsive protein crosslinkers.

### 3.5. Folding/Unfolding

Protein structures consist of α-helices and/or β-sheets stabilized by hydrogen bonds and hydrophobic, electrostatic interactions. Extreme temperature, chemical denaturants, etc., can trigger protein unfolding which generates the ultimate structural transition which is more significant than conventional conformational changes. A subset of responsive hydrogels can be prepared through protein folding/unfolding. External stimuli such as denaturants and temperature cause protein unfolding and significantly diminish the mechanical strength of the hydrogels. After removing these stimuli, the protein refolds and restores the mechanical strength of the hydrogel [49]. Recently, the Li group constructed a thermo-responsive hydrogel by using a hydrophobic peptide–hydrophilic protein copolymer that could undergo heat-induced reversible folding and unfolding of the hydrogel, resulting in altered mechanical properties [25]. Li Group utilized a mutually exclusive protein (MEP) as a scaffold to achieve the folding and unfolding switch. In this system, a guest domain with a long end-to-end distance was fused with a short loop of a host domain, creating a folding conflict between the host and guest domains due to their topological constrains. In this situation, only one of the two domains can fold at a given time. By employing the MEP system, diverse moieties that respond to ion strength, metal ions and redox environments can be introduced. For example, by introducing a metal (Ni^2+^) chelation motif or di-cysteine mutations into the host domain, the Ni^2+^–histidine interaction or disulfide bond formation could trigger the shift of the conformational equilibrium of MEP, leading to significant changes in mechanical and physical properties [29]. This strategy sheds new light on the design of dynamic protein hydrogels with shape-morphing/memory properties [48].

## 4. Discussion

The properties of stimuli-responsive hydrogels make them suitable for a wide range of applications, including drug delivery, tissue engineering, and biosensors [90]. Protein-based responsive hydrogels, which can change their physical and mechanical properties in response to external stimuli, have attracted increasing attention in the past decades. By taking the advantages of DNA-recombination technology and protein engineering, researchers can design novel protein-based hydrogels that bear remarkable mechanical properties and environmental responsiveness. Compared with traditional hydrogels, protein-based responsive hydrogels offer enhanced biocompatibility and biodegradability, making them highly suitable for a range of biomedical applications including tissue engineering and regenerative medicine. The ability to sense specific stimuli allows for more precise control over the gel properties and behavior, leading to improved functionality for various applications. Additionally, these hydrogels can be engineered to release drugs or therapeutic agents in response to specific triggers in the body, enabling targeted and controlled drug delivery with reduced side effects. However, protein-based hydrogels may exhibit lower mechanical strength and stability, which can limit their use in certain load-bearing applications compared to traditional hydrogels. The fabrication of stimuli-responsive protein hydrogels with tailored responsiveness requires intricate design and engineering, which can be time-consuming and more expensive than synthetic hydrogels. Further works and developments are required to fully explore and enhance their capabilities, including improving the specificity through precise control over responsiveness, expanding the scope of responsiveness, developing multi-responsive protein hydrogels, and employing artificial intelligence for engineering protein-responsive hydrogels.

### 4.1. Precise Control over Responsiveness

One of the primary challenges of the clinical application of responsive protein hydrogels is achieving precise control over their responsiveness. The majority of protein hydrogels offer poor control over drug release, and achieving fine-tuned and graded responses remains a challenge. The cross-linking density and length of the hydrogel plays a crucial role in its mechanical properties and responsiveness. Higher cross-linking densities generally lead to stiffer and less responsive hydrogels, while lower densities result in softer and more responsive hydrogels. By adjusting the cross-linking density or length, the hydrogel’s mechanical properties and responsiveness can be controlled. Furthermore, different types of protein–ligand interactions and intensities offer great opportunities for protein-based hydrogels to achieve precise control over ligand stimuli. This enables the development of hydrogels with tailored responses and functionalities for various applications in biomedicine.

### 4.2. Expanding the Responsive Scope

Given the fast developments in designing synthetic heteropolymers and the huge library of proteins, it is expected that more types of proteins that display conformational changes or folding/unfolding transitions to external stimuli can be integrated into hydrogel networks for constructing responsive protein hydrogels. Moreover, a variety of chemical reactions can be combined with biorecognition to transform nanoscale conformational changes into macroscopic motion. Some stimuli-responsive hydrogels can sense strict temperature and ultraviolet light, but these properties may not be suitable for *in vivo* applications. Therefore, for thermal-responsive hydrogels, it is suitable to develop protein hydrogels with temperature sensitivity around near-body-temperature to ensure biocompatibility. For light-responsive hydrogels, the current UV- or blue light-responsive protein hydrogels are limited for clinical applications due to their cytotoxicity and limited tissue penetration. It is highly desirable to expand the use of NIR-responsive hydrogels, which offer low phototoxicity and enhanced penetration depth, making them more suitable for medical applications.

Magnets exhibit the advantages of fast-response and remote-control properties, making magnetic-responsive hydrogel an ideal biomaterial for clinical application. The currently reported protein-based magnetic responsive hydrogels were constructed by directly embedding magnetic nanoparticles such as Fe_3_O_4_ as a dispersion in protein hydrogel matrices [91]. It is highly desirable to integrate functional magnetic-responsive moieties into the protein hydrogel network. The development of magnetic-responsive proteins with conformational changes may provide a new avenue for magnetic-responsive protein hydrogel construction.

Apart from conventional physical and chemical stimuli, the specific and versatile features of protein–ligand interactions can be harnessed into protein-based responsive hydrogels. This can involve the use of specific binding domains of proteins that interact with its recognition substrates. This recognition can serve as a controllable crosslinker that can selectively respond to specific ligands, providing an accurate regulation over its responsiveness. Advancements in this area have the potential to unlock greater possibilities for these innovative materials in addressing complex biomedical challenges.

### 4.3. Multi-Responsive Protein Hydrogel

Proteins with versatile responsive properties can be genetically fused together to create multi-functional proteins and thus integrated into polymeric hydrogel networks for sophisticated regulation in response to cellular environmental stimuli. The versatile nature of multi-responsive protein hydrogels offers a range of possibilities for on-demand drug release, personalized medicine, and advanced wound healing therapies. For example, dual-responsive hydrogels, capable of simultaneously responding to changes in temperature and pH, can be designed from thermo-and pH-responsive biopolymers, respectively, for potential drug delivery applications. Kim et al. reported a multi-responsive hydrogel using a hybrid protein that combines domains of spider silk, amyloid, and mussel foot protein (Mfp). The innovative hydrogel, rich in large number of β-crystals from the silk protein, displays a high stretchability and toughness, and is adhesive to a wide range of surfaces due to the presence of the Mfp sequence. The oxidative stimuli attenuated the intermolecular interactions within the hydrogel, resulting in changes in cohesive strength. This selective debonding can be blocked through the addition of Fe^3+^ ions [92]. The multi-responsiveness allows for a high degree of control over the hydrogel properties, which can be adjusted according to the specific needs of the application. Further development in this field is expected to investigate novel techniques for incorporating multiple stimuli-responsive domains and for precisely adjusting the multi-responsive characteristics of these hydrogels to broaden their potential applications in the fields of biomedical and biotechnology.

### 4.4. AI-Assisted Responsive Protein Hydrogel

The optimization of responsive hydrogel properties involves the screening of suitable building blocks of hydrogel and the tuning of crosslinker density and/or length. This process is cost and labor extensive. For example, to achieve the desired self-assembly characteristics of SELP-based hydrogels, the ratio of silk-to-elastin blocks in a SELP building block needs to be fine-tuned through high-throughput synthesis and screening. It is highly demanded to develop more quantitative theories and methods to bridge the microscopic properties of building blocks to the macroscopic properties of hydrogels. With the fast advancement of machine learning algorithms, AI-driven hydrogel design can optimize the ideal combination of hydrogel building blocks and crosslinkers to predict how a hydrogel will swell, degrade, or release drugs in response to changes in pH, temperature, or biological factors. Through the use of genetic algorithms and Bayesian optimization, AI-facilitated screening can be used for searching for optimal hydrogel building blocks and crosslinkers within vast chemical and protein structural spaces. These techniques would enable the evaluation of vast libraries of hydrogel formulations with different responsiveness, accelerating the development of hydrogels tailored for desirable properties which can be applied for wound healing, drug releasing, or tissue engineering.

## 5. Conclusions

Protein-based hydrogels with stimuli-responsive functionalities have emerged as promising biomaterials for a wide range of biomedical applications. By integrating engineered proteins with physical-, chemical-, and biological-responsive moieties, researchers have been able to develop dynamic hydrogels that can respond to environmental stimuli. This review has summarized the current advances in responsive protein hydrogels, highlighting their design approaches and responsive properties upon various stimuli. The key responsive units and cross-linking strategies that permit protein hydrogels to exhibit stimuli responsiveness were also discussed. Furthermore, it addressed the current challenges and future perspectives in this field, emphasizing the need for further research and development efforts. With continued research and development, responsive protein hydrogels are poised to play a pivotal role in shaping the future of biomaterials for medical application, holding great potential to open up new avenues for personalized and targeted therapeutic solutions.

## Figures and Tables

**Table 1:** Cross-linking strategies for stimuli-responsive protein hydrogels.

Type of Stimuli	Stimuli-Response Mode	Cases
Light	Oligomerization switch of photoreceptors induced by light	Cph1 [9], PhyB-PIF6 [14], LOV2-zdk1 [15], CarHc [16,17], UVR8 [18], Dronpa145N [19,20]
	Photo cleavage	PhoCl [21,22]
Temperature	Temperature-induced phase transition	Tannic-gelatin [23], Q protein [10], ELPs and ELP-derived peptides [13,24,25,26], γ-glutamic-collagen [27],
	Non-covalent protein assembly	CTD [28]
Metal ion	Metal coordination with histidine residues	His-tag coordinate with Ni^2+^ [29,30,31], Co^2+^ [32], Zn^2+^ [33,34]
	Ca^2+^-induced protein self-assembly	Ca^2+^-dependent CaM-ligand M13-peptide binding [35,36]
Redox	Inter-and-intra cysteine-disulfide transition	Disulfide bonds in Resilin [11], SELPs [37], BSA [38,39], keratin [40,41], SF [42], CLP [43], ELP [44]
	Disulfide bond mediated assembly of split protein	Split G_N_/G_C_ [45]
pH and Ionic strength	Reversible Schiff base dynamic change	Schiff base reactions between aldehyde and amine groups in proteins [46,47]
	The protein domain fold and unfold	(GB1)8 and (FL)8 [48]
	Coiled-coil interactions	BSA [49]
	β-Sheet Assembly	Shell nacre protein (r-n 16.3) [50]
Enzyme	Enzyme catalyzed crosslinker cleavage	MMP7 [51,52,53], TP [54]
Ligand	Ligands binding with receptor	Aktm/ATP [55] Novobiocin/coumermycin-GyrB [56] CaM/Phenothiazine or trifluowere [57] Maltose-MBP [12,58]

## Data Availability

Not applicable.

## References

[1] Vashist A., Kaushik A., Vashist A., Sagar V., Ghosal A., Gupta Y.K., Ahmad S., Nair M. (2018). Advances in Carbon Nano-tubes-Hydrogel Hybrids in Nanomedicine for Therapeutics. Adv. Healthc. Mater.

[2] Bae S.-W., Kim J., Kwon S. (2022). Recent Advances in Polymer Additive Engineering for Diagnostic and Therapeutic Hydrogels. Int. J. Mol. Sci..

[3] Yadav N., Chauhan M.K., Chauhan V.S. (2020). Short to Ultrashort Peptide-Based Hydrogels as a Platform for Biomedical Appli-cations. Biomater. Sci..

[4] Davari N., Bakhtiary N., Khajehmohammadi M., Sarkari S., Tolabi H., Ghorbani F., Ghalandari B. (2022). Protein-Based Hydrogels: Promising Materials for Tissue Engineering. Polymers.

[5] Chander S., Kulkarni G.T., Dhiman N., Kharkwal H. (2021). Protein-Based Nanohydrogels for Bioactive Delivery. Front. Chem..

[6] Cao H., Duan L., Zhang Y., Cao J., Zhang K. (2021). Current Hydrogel Advances in Physicochemical and Biological Re-sponse-Driven Biomedical Application Diversity. Signal Transduct Tar..

[7] Deptuch T., Penderecka K., Kaczmarek M., Molenda S., Dams-Kozlowska H. (2022). In vivo study of the immune response to bioengineered spider silk spheres. Sci. Rep..

[8] Li Y., Xue B., Cao Y. (2020). 100th Anniversary of Macromolecular Science Viewpoint: Synthetic Protein Hydrogels. ACS Macro Lett..

[9] Hörner M., Raute K., Hummel B., Madl J., Creusen G., Thomas O.S., Christen E.H., Hotz N., Gübeli R.J., Engesser R. (2019). Phytochrome-Based Extracellular Matrix with Reversibly Tunable Mechanical Properties. Adv. Mater..

[10] Hill L.K., Meleties M., Katyal P., Xie X., Delgado-Fukushima E., Jihad T., Liu C.-F., O’neill S., Tu R.S., Renfrew P.D. (2019). Thermoresponsive Protein-Engineered Coiled-Coil Hydrogel for Sustained Small Molecule Release. Biomacromolecules.

[11] Qin G., Rivkin A., Lapidot S., Hu X., Preis I., Arinus S.B., Dgany O., Shoseyov O., Kaplan D.L. (2011). Recombinant exon-encoded resilins for elastomeric biomaterials. Biomaterials.

[12] Hughes M.D.G., Cussons S., Mahmoudi N., Brockwell D.J., Dougan L. (2022). Tuning Protein Hydrogel Mechanics through Mod-ulation of Nanoscale Unfolding and Entanglement in Postgelation Relaxation. ACS Nano.

[13] Dai M., Goudounet G., Zhao H., Garbay B., Garanger E., Pecastaings G., Schultze X., Lecommandoux S. (2020). Thermosensitive Hybrid Elastin-like Polypeptide-Based ABC Triblock Hydrogel. Macromolecules.

[14] Beyer H.M., Thomas O.S., Riegel N., Zurbriggen M.D., Weber W., Hörner M. (2018). Generic and reversible opto-trapping of biomolecules. Acta Biomater..

[15] Duan T., Bian Q., Li H. (2021). Light-Responsive Dynamic Protein Hydrogels Based on LOVTRAP. Langmuir.

[16] Wang R., Yang Z., Luo J., Hsing I.-M., Sun F. (2017). B_12_–dependent photoresponsive protein hydrogels for controlled stem cell/protein release. Proc. Natl. Acad. Sci. USA.

[17] Narayan O.P., Mu X., Hasturk O., Kaplan D.L. (2020). Dynamically tunable light responsive silk-elastin-like proteins. Acta Biomater..

[18] Zhang X., Dong C., Huang W., Wang H., Wang L., Ding D., Zhou H., Long J., Wang T., Yang Z. (2015). Rational design of a photo-responsive UVR8-derived protein and a self-assembling peptide–protein conjugate for responsive hydrogel formation. Nanoscale.

[19] Lyu S., Fang J., Duan T., Fu L., Liu J., Li H. (2017). Optically controlled reversible protein hydrogels based on photoswitchable fluorescent protein Dronpa. Chem. Commun..

[20] Wu X., Huang W., Wu W.-H., Xue B., Xiang D., Li Y., Qin M., Sun F., Wang W., Zhang W.-B. (2017). Reversible hydrogels with tunable mechanical properties for optically controlling cell migration. Nano Res..

[21] Shadish J.A., Strange A.C., DeForest C.A. (2019). Genetically Encoded Photocleavable Linkers for Patterned Protein Release from Biomaterials. J. Am. Chem. Soc..

[22] Xiang D., Wu X., Cao W., Xue B., Qin M., Cao Y., Wang W. (2020). Hydrogels with Tunable Mechanical Properties Based on Photocleavable Proteins. Front. Chem..

[23] Yang S., Zhang Y., Wang T., Sun W., Tong Z. (2020). Ultrafast and Programmable Shape Memory Hydrogel of Gelatin Soaked in Tannic Acid Solution. ACS Appl. Mater. Interfaces.

[24] Parker R.N., Cairns D.M., Wu W.A., Jordan K., Guo C., Huang W., Martin-Moldes Z., Kaplan D.L. (2020). Smart Material Hy-drogel Transfer Devices Fabricated with Stimuli-Responsive Silk-Elastin-Like Proteins. Adv. Healthc. Mater.

[25] Duan T., Li H. (2020). In Situ Phase Transition of Elastin-Like Polypeptide Chains Regulates Thermoresponsive Properties of Elas-tomeric Protein-Based Hydrogels. Biomacromolecules.

[26] Song W.-W., Qian Z.-G., Liu H., Chen H.-F., Kaplan D.L., Xia X.-X. (2021). On-Demand Regulation of Dual Thermosensitive Protein Hydrogels. ACS Macro Lett..

[27] Cho S.-H., Kim A., Shin W., Heo M.B., Noh H.J., Hong K.S., Cho J.-H., Lim Y.T. (2017). Photothermal-modulated drug delivery and magnetic relaxation based on collagen/poly(γ-glutamic acid) hydrogel. Int. J. Nanomed..

[28] Qian Z.-G., Zhou M.-L., Song W.-W., Xia X.-X. (2015). Dual Thermosensitive Hydrogels Assembled from the Conserved C-Terminal Domain of Spider Dragline Silk. Biomacromolecules.

[29] Kong N., Li H. (2016). Rationally Designed Dynamic Protein Hydrogels with Reversibly Tunable Mechanical Properties. Biophys. J..

[30] Wang Y., Wang X., Montclare J.K. (2021). Effect of Divalent Metal Cations on the Conformation, Elastic Behavior, and Controlled Release of a Photocrosslinked Protein Engineered Hydrogel. ACS Appl. Bio Mater..

[31] Kong N., Fu L., Peng Q., Li H. (2016). Metal Chelation Dynamically Regulates the Mechanical Properties of Engineered Protein Hydrogels. ACS Biomater. Sci. Eng..

[32] Kou S., Yang X., Yang Z., Liu X., Wegner S.V., Sun F. (2019). Cobalt-Cross-Linked, Redox-Responsive Spy Network Protein Hy-drogels. ACS Macro Lett..

[33] Jiang B., Liu X., Yang C., Yang Z., Luo J., Kou S., Liu K., Sun F. (2020). Injectable, photoresponsive hydrogels for delivering neuroprotective proteins enabled by metal-directed protein assembly. Sci. Adv..

[34] Chen X., Tan B., Wang S., Tang R., Bao Z., Chen G., Chen S., Tang W., Wang Z., Long C. (2021). Rationally designed protein cross-linked hydrogel for bone regeneration via synergistic release of magnesium and zinc ions. Biomaterials.

[35] Luo J., Sun F. (2020). Calcium-responsive hydrogels enabled by inducible protein–protein interactions. Polym. Chem..

[36] Gallivan J.P., Topp S. (2004). Engineering of Stimuli-Sensitive Biomaterials That Respond to Calcium Ions. Abstr. Pap. Am. Chem. S.

[37] Zhou M.-L., Qian Z.-G., Chen L., Kaplan D.L., Xia X.-X. (2016). Rationally Designed Redox-Sensitive Protein Hydrogels with Tunable Mechanical Properties. Biomacromolecules.

[38] Nikfarjam S., Gibbons R., Burni F., Raghavan S.R., Anisimov M.A., Woehl T.J. (2023). Chemically Fueled Dissipative Cross-Linking of Protein Hydrogels Mediated by Protein Unfolding. Biomacromolecules.

[39] Jiao C., Obst F., Geisler M., Che Y., Richter A., Appelhans D., Gaitzsch J., Voit B. (2022). Reversible Protein Capture and Release by Redox-Responsive Hydrogel in Microfluidics. Polymers.

[40] Cao Y., Yao Y., Li Y., Yang X., Cao Z., Yang G. (2019). Tunable keratin hydrogel based on disulfide shuffling strategy for drug delivery and tissue engineering. J. Colloid Interface Sci..

[41] Ham T.R., Lee R.T., Han S., Haque S., Vodovotz Y., Gu J., Burnett L.R., Tomblyn S., Saul J.M. (2015). Tunable Keratin Hydrogels for Controlled Erosion and Growth Factor Delivery. Biomacromolecules.

[42] Mcgill M., Grant J.M., Kaplan D.L. (2020). Enzyme-Mediated Conjugation of Peptides to Silk Fibroin for Facile Hydrogel Function-alization. Ann. Biomed. Eng..

[43] Jia S., Wang J., Wang X., Liu X., Li S., Li Y., Li J., Wang J., Man S., Guo Z. (2023). Genetically encoded *in situ* gelation redox-responsive collagen-like protein hydrogel for accelerating diabetic wound healing. Biomater. Sci..

[44] Asai D., Xu D., Liu W., Quiroz F.G., Callahan D.J., Zalutsky M.R., Craig S.L., Chilkoti A. (2012). Protein polymer hydrogels by in situ, rapid and reversible self-gelation. Biomaterials.

[45] Wang R., Li J., Li X., Guo J., Liu J., Li H. (2019). Engineering protein polymers of ultrahigh molecular weight via supramolecular polymerization: Towards mimicking the giant muscle protein titin. Chem. Sci..

[46] Fu F., Chen Z., Zhao Z., Wang H., Shang L., Gu Z., Zhao Y. (2017). Bio-inspired self-healing structural color hydrogel. Proc. Natl. Acad. Sci. USA.

[47] Yuan T., Fei J., Xu Y., Yang X., Li J. (2017). Stimuli-Responsive Dipeptide–Protein Hydrogels through Schiff Base Coassembly. Macromol. Rapid Comm..

[48] Bian Q., Fu L., Li H. (2022). Engineering shape memory and morphing protein hydrogels based on protein unfolding and folding. Nat. Commun..

[49] Khoury L.R., Popa I. (2019). Chemical unfolding of protein domains induces shape change in programmed protein hydrogels. Nat. Commun..

[50] Perovic I., Davidyants A., Evans J.S. (2016). Aragonite-Associated Mollusk Shell Protein Aggregates To Form Mesoscale “Smart” Hydrogels. ACS Omega.

[51] Parmar P.A., St-Pierre J.-P., Chow L.W., Spicer C.D., Stoichevska V., Peng Y.Y., Werkmeister J.A., Ramshaw J.A., Stevens M.M. (2017). Enhanced articular cartilage by human mesenchymal stem cells in enzymatically mediated transiently RGDS-functionalized collagen-mimetic hydrogels. Acta Biomater..

[52] Parmar P.A., Skaalure S.C., Chow L.W., St-Pierre J.-P., Stoichevska V., Peng Y.Y., Werkmeister J.A., Ramshaw J.A., Stevens M.M. (2016). Temporally degradable collagen–mimetic hydrogels tuned to chondrogenesis of human mesenchymal stem cells. Biomaterials.

[53] Parmar P.A., Chow L.W., St-Pierre J.P., Horejs C.M., Peng Y.Y., Werkmeister J.A., Ramshaw J.A., Stevens M.M. (2015). Colla-gen-Mimetic Peptide-Modifiable Hydrogels for Articular Cartilage Regeneration. Biomaterials.

[54] Ji Y.-R., Hsu Y.-H., Syue M.-H., Wang Y.-C., Lin S.-Y., Huang T.-W., Young T.-H. (2022). Controlled Decomposable Hydrogel Triggered with a Specific Enzyme. ACS Omega.

[55] Yuan W., Yang J., Kopeckova P., Kopecek J. (2008). Smart Hydrogels Containing Adenylate Kinase: Translating Substrate Recog-nition into Macroscopic Motion. J. Am. Chem. Soc..

[56] Ehrbar M., Schoenmakers R., Christen E.H., Fussenegger M., Weber W. (2008). Drug-sensing hydrogels for the inducible release of biopharmaceuticals. Nat. Mater..

[57] King W.J., Pytel N.J., Ng K., Murphy W.L. (2010). Triggered Drug Release from Dynamic Microspheres via a Protein Conformational Change. Macromol. Biosci..

[58] Hughes M.D.G., Cussons S., Mahmoudi N., Brockwell D.J., Dougan L. (2020). Single molecule protein stabilisation translates to macromolecular mechanics of a protein network. Soft Matter.

[59] Zhou X.X., Chung H.K., Lam A.J., Lin M.Z. (2012). Optical Control of Protein Activity by Fluorescent Protein Domains. Science.

[60] Wu D., Hu Q., Yan Z., Chen W., Yan C., Huang X., Zhang J., Yang P., Deng H., Wang J. (2012). Structural Basis of Ultra-violet-B Perception by Uvr8. Nature.

[61] Hammer J.A., Ruta A., West J.L. (2020). Using Tools from Optogenetics to Create Light-Responsive Biomaterials: Lovtrap-Peg Hy-drogels for Dynamic Peptide Immobilization. Ann. Biomed. Eng..

[62] Zhang W., Lohman A.W., Zhuravlova Y., Lu X., Wiens M.D., Hoi H., Yaganoglu S., A Mohr M., Kitova E.N., Klassen J.S. (2017). Optogenetic control with a photocleavable protein, PhoCl. Nat. Methods.

[63] Westerlund A.M., Delemotte L. (2018). Effect of Ca2+ on the promiscuous target-protein binding of calmodulin. PLoS Comput. Biol..

[64] Zhang X., Jiang S., Yan T., Fan X., Li F., Yang X., Ren B., Xu J., Liu J. (2019). Injectable and fast self-healing protein hydrogels. Soft Matter.

[65] Su R.S., Galas R.J., Lin C., Liu J.C. (2019). Redox-Responsive Resilin-Like Hydrogels for Tissue Engineering and Drug Delivery Applications. Macromol. Biosci..

[66] Longo G.S., Pérez-Chávez N.A., Szleifer I. (2019). How Protonation Modulates the Interaction between Proteins and Ph-Responsive Hydrogel Films. Curr. Opin. Colloid Interface Sci..

[67] Kimura S., Komiyama T., Masuzawa T., Yokoya M., Oyoshi T., Yamanaka M. (2023). Bovine Serum Albumin Hydrogel Formation: pH Dependence and Rheological Analyses. Chem. Pharm. Bull..

[68] Ren Y., Yu X., Li Z., Liu D., Xue X. (2020). Fabrication of Ph-Responsive Ta-Keratin Bio-Composited Hydrogels Encapsulated with Photoluminescent Go Quantum Dots for Improved Bacterial Inhibition and Healing Efficacy in Wound Care Management: In Vivo Wound Evaluations. J. Photoch. Photobio B.

[69] Zhang R., Tian Y., Pang L., Xu T., Yu B., Cong H., Shen Y. (2022). Wound Microenvironment-Responsive Protein Hydrogel Drug-Loaded System with Accelerating Healing and Antibacterial Property. ACS Appl. Mater. Interfaces.

[70] Ponce C.B., Evans J.S. (2011). Polymorph Crystal Selection by n16, an Intrinsically Disordered Nacre Framework Protein. Cryst. Growth Des..

[71] He Q., Huang Y., Wang S. (2017). Hofmeister Effect-Assisted One Step Fabrication of Ductile and Strong Gelatin Hydrogels. Adv. Funct. Mater..

[72] Jiang L., Su D., Ding S., Zhang Q., Li Z., Chen F., Ding W., Zhang S., Dong J. (2019). Salt-Assisted Toughening of Protein Hydrogel with Controlled Degradation for Bone Regeneration. Adv. Funct. Mater..

[73] Hu C., Long L., Cao J., Zhang S., Wang Y. (2021). Dual-Crosslinked Mussel-Inspired Smart Hydrogels with Enhanced Antibacterial and Angiogenic Properties for Chronic Infected Diabetic Wound Treatment Via Ph-Responsive Quick Cargo Release. Chem. Eng. J..

[74] Qu X., Yang Z. (2016). Benzoic-Imine-Based Physiological-Ph-Responsive Materials for Biomedical Applications. Chem. Asian J..

[75] Hardy J.G., Lin P., Schmidt C.E. (2015). Biodegradable Hydrogels Composed of Oxime Crosslinked Poly(Ethylene Glycol), Hyaluronic Acid and Collagen: A Tunable Platform for Soft Tissue Engineering. J. Biomater. Sci. Polym. Ed.

[76] Fang J., Mehlich A., Koga N., Huang J., Koga R., Gao X., Hu C., Jin C., Rief M., Kast J. (2013). Forced protein unfolding leads to highly elastic and tough protein hydrogels. Nat. Commun..

[77] Sui Z., King W.J., Murphy W.L. (2007). Dynamic Materials Based on a Protein Conformational Change. Adv. Mater..

[78] Zakeri B., Fierer J.O., Celik E., Chittock E.C., Schwarz-Linek U., Moy V.T., Howarth M. (2012). Peptide tag forming a rapid covalent bond to a protein, through engineering a bacterial adhesin. Proc. Natl. Acad. Sci. USA.

[79] Arkenberg M.R., Moore D.M., Lin C.C. (2019). Dynamic Control of Hydrogel Crosslinking Via Sortase-Mediated Reversible Trans-peptidation. Acta Biomater..

[80] Shadish J.A., Benuska G.M., DeForest C.A. (2019). Bioactive site-specifically modified proteins for 4D patterning of gel biomaterials. Nat. Mater..

[81] Mao H., Hart S.A., Schink A., Pollok B.A. (2004). Sortase-Mediated Protein Ligation: A New Method for Protein Engineering. J. Am. Chem. Soc..

[82] Ramirez M.A., Chen Z. (2017). Split-Intein Triggered Protein Hydrogels. Methods Mol. Biol..

[83] Andresen M., Stiel A.C., Trowitzsch S., Weber G., Eggeling C., Wahl M.C., Hell S.W., Jakobs S. (2007). Structural Basis for Re-versible Photoswitching in Dronpa. Proc. Natl. Acad. Sci. USA.

[84] Wang H., Vilela M., Winkler A., Tarnawski M., Schlichting I., Yumerefendi H., Kuhlman B., Liu R., Danuser G., Hahn K.M. (2016). LOVTRAP: An optogenetic system for photoinduced protein dissociation. Nat. Methods.

[85] Nijenhuis K.T. (2006). On the nature of crosslinks in thermoreversible gels. Polym. Bull..

[86] Yan R., Liu X., Xiong J., Feng Q., Xu J., Wang H., Xiao K. (2020). Ph-Responsive Hyperbranched Polypeptides Based on Schiff Bases as Drug Carriers for Reducing Toxicity of Chemotherapy. RSC Adv..

[87] Tang A., Li Y., Yao Y., Yang X., Cao Z., Nie H., Yang G. (2021). Injectable keratin hydrogels as hemostatic and wound dressing materials. Biomater. Sci..

[88] Chou C.-C., Martin-Martinez F.J., Qin Z., Dennis P.B., Gupta M.K., Naik R.R., Buehler M.J. (2017). Ion Effect and Met-al-Coordinated Cross-Linking for Multiscale Design of Nereis Jaw Inspired Mechanomutable Materials. ACS Nano.

[89] Sahajpal K., Shekhar S., Kumar A., Sharma B., Meena M.K., Bhagi A.K., Sharma S. (2022). Dynamic protein and polypeptide hydrogels based on Schiff base co-assembly for biomedicine. J. Mater. Chem. B.

[90] Shymborska Y., Budkowski A., Raczkowska J., Donchak V., Melnyk Y., Vasiichuk V., Stetsyshyn Y. (2023). Switching it Up: The Promise of Stimuli-Responsive Polymer Systems in Biomedical Science. Chem. Rec..

[91] Hu S.-H., Liu T.-Y., Liu D.-M., Chen S.-Y. (2007). Nano-ferrosponges for controlled drug release. J. Control. Release.

[92] Kim E., Jeon J., Zhu Y., Hoppe E.D., Jun Y.-S., Genin G.M., Zhang F. (2021). A Biosynthetic Hybrid Spidroin-Amyloid-Mussel Foot Protein for Underwater Adhesion on Diverse Surfaces. ACS Appl. Mater. Interfaces.

